# Editorial

**DOI:** 10.1120/jacmp.v6i1.2113

**Published:** 2005-03-17

The JACMP, which has completed five years of publication, still stands as the only journal that publishes exclusively clinical and professional articles for the medical physics community. Since its inception in 1999, the goal of our Board of Editors has been to maintain the clinical and practical focus of the Journal. We wish to remind our readers, contributors, and reviewers of the scope of articles considered for publication by the JACMP:

1. Articles that describe a unique solution to a clinical problem for a specific patient, type of patient target volume, or piece of equipment;
2. Equipment evaluation and review articles describing commissioning tests and techniques for clinical implementation;
3. Articles that describe multiple types of equipment, often from different vendors, that must work together and communicate seamlessly;
4. Health physics papers describing unique shielding or radiation protection problems;
5. Papers dealing with quality assurance, continuing quality improvement, and total quality management;
6. Articles dealing with administrative problems including staffing, space, equipment evaluation and purchase, project analysis, and resource planning;
7. Review articles dealing with how different institutions and vendors approach similar clinical problems;
8. Articles describing ways to implement and verify recommendations from scientific bodies (e.g., task groups) while recognizing specific equipment or clinical limitations;
9. Business management articles addressing the changing reimbursement and management landscape and how individual medical physicists and the medical physics community might be proactive;
10. Reviews of emerging technologies and changing regulatory standards and practice accreditation;
11. Debates about clinical issues;
12. Medical‐legal issues that impact the practice of medical physics.

The Editorial Board of the Journal is responsible for establishing the list of article types to be included in the Journal. The above list was approved by the initial Board of Editors in 1999 and has not changed since its inception. Please note that nonclinical medical physics science articles should be considered by a journal specializing in these areas. Such journals include *Medical Physics,* published in the United States, and *Physics in Medicine and Biology,* published in Great Britain. The JACMP is pleased to consider clinical articles of high scientific merit within the scope of the areas of inquiry outlined above.

The Editorial Board is eager to receive practical clinical articles on how to provide the best professional care for radiology and radiation oncology patients. We encourage our contributors by providing careful guidance and reviews that benefit medical physicists and their patients. We are pleased to be working with you.

Michael D. Mills, Ph.D.,

Editor‐in‐Chief, JACMP

## The Journal of Applied Clinical Medical Physics

**Table:** 

Editorial Board 2005	
Editor‐in‐Chief	Michael D. Mills
Deputy Editor‐in‐Chief	Timothy D. Solberg
Associate Editor‐at‐Large	Richard Stark
**Associate Editors**	
Radiation Oncology Physics	Nzhde Agazaryan, B. Gino Fallone,
	John Gibbons, Michael Herman,
	Edwin McCullough, Janelle Molloy,
	Matthew Podgorsak, Nikos Papanikolaou,
	Mehrdad Sarfaraz, Lu Wang
Medical Imaging	Walter Huda, William Pavlicek
Radiation Measurements	Larry DeWerd
Radiation Protection & Regulations	James Deye
Nonionizing Topics	Bhudatt Paliwal
Other Topics	Timothy Solberg
Book/Media Reviews	James Smathers
Editorials	John Horton
**Journal Production Team**	
Manuscript Manager	Patricia Walker
Copy Editor	Betty R. Robinson
Layout Editor	Melanie Marques
Proofreader	Heather Shand

